# A Pilot Study on the Effects of l-Carnitine and Trimethylamine-N-Oxide on Platelet Mitochondrial DNA Methylation and CVD Biomarkers in Aged Women

**DOI:** 10.3390/ijms21031047

**Published:** 2020-02-05

**Authors:** Laura Bordoni, Angelika K. Sawicka, Arkadiusz Szarmach, Pawel J. Winklewski, Robert A. Olek, Rosita Gabbianelli

**Affiliations:** 1 School of Pharmacy, Unit of Molecular Biology, University of Camerino, 62032 Camerino, Italy; rosita.gabbianelli@unicam.it; 2 Doctoral School for Physical Culture Sciences, 80-336 Gdansk, Poland; ange.sawicka@gmail.com; 3 Department of Human Physiology, Faculty of Health Sciences, Medical University of Gdansk, 80-210 Gdansk, Poland; pawelwinklewski@wp.pl; 4 2nd Department of Radiology, Faculty of Health Sciences, Medical University of Gdansk, 80-210 Gdansk, Poland; a.szarmach@gumed.edu.pl; 5 Department of Athletics, Strength and Conditioning, Poznan University of Physical Education, 61-871 Poznan, Poland; robert.olek@aol.com

**Keywords:** mtDNA methylation, TMAO, D-loop, CVD, l-carnitine, nutrigenomics, biomarker

## Abstract

l-carnitine supplementation has been used for cardiovascular health protection for a long time. Recently, trimethylamine-N-oxide (TMAO), which is an end product of l-carnitine metabolism via the activity of microbiota, has been identified as a cardiovascular disease (CVD) biomarker. The aim of this study was to assess the effect of 6 months of l-carnitine supplementation in a group of aged women engaged in a regular physical training. Platelet mitochondrial DNA methylation, an emerging and innovative biomarker, lipid profile and TMAO levels have been measured. TMAO increased after l-carnitine supplementation (before 344.3 ± 129.8 ng/mL vs. after 2216.8 ± 1869.0 ng/mL; *n* = 9; paired t-test, *p* = 0.02). No significant effects on TMAO were exerted by training alone (*n* = 9) or by l-leucine supplementation (*n* = 12). TMAO levels after 6 months of l-carnitine supplementation were associated with higher low-density lipoprotein-cholesterol (LDL-c) (Spearman Rho = 0.518, *p* = 0.003) and total cholesterol (TC) (Spearman Rho = 0.407, *p* = 0.026) levels. l-carnitine supplementation increased D-loop methylation in platelets (+6.63%; paired t-test, *p* = 0.005). D-loop methylation was not directly correlated to the TMAO augmentation observed in the supplemented group, but its increase inversely correlated with TC (Pearson coefficient = −0.529, *p* = 0.029) and LDL-c (Pearson coefficient = −0.439, *p* = 0.048). This evidence supports the hypothesis that the correlation between l-carnitine, TMAO and atherosclerosis might be more complex than already postulated, and the alteration of mitochondrial DNA (mtDNA) methylation in platelets could be involved in the pathogenesis of this multifactorial disease.

## 1. Background

Cardiovascular disease (CVD) is a leading cause of mortality worldwide and it produces major burdens on morbidity, quality of life, and social costs [1]. Among several other aspects, nutrition has been indexed as among the most effective CVD preventive factors [2,3,4]. Indeed, evidence suggests that dietary nutrients can play a role in the onset and development of atherosclerosis. A particular interest is currently directed to phosphatidylcholine, choline and l-carnitine [5,6,7], because these compounds can be metabolized by gut microflora in trimethylamine (TMA), which, in turn, can be converted in the liver by the host flavin monooxygenases into TMA-N-oxide (TMAO) [8]. Recent systematic reviews and meta-analyses demonstrated a positive correlation between elevated plasma TMAO and an increased risk for major adverse cardio and cerebrovascular events [9,10], suggesting a TMAO contribution in CVD etiology. Several mechanisms have been proposed to be involved, such as alteration of cholesterol homeostasis [11], promotion of vascular inflammation [12], but also impairment of methyl metabolism [13]. It has also been demonstrated that the direct exposure of platelets to TMAO boosts platelet activation [14]. In this context, it is interesting to notice that platelet mitochondrial DNA (mtDNA) methylation has been recently proposed as a new molecular biomarker of cardiovascular health [15]. Epigenetic alterations (in particular DNA methylation) have been previously reported to play a role in CVD [16,17], gaining increasing interest in view of potential development of new therapeutic approaches [18,19], more efficient tools for early diagnosis [20] and personalized interventions [21]. While epigenetic regulation of nuclear DNA has been widely investigated, a relatively new field of research is represented by mitochondrial epigenetics [22,23,24,25]. Several studies identified variations of methylation levels in different areas of the mitochondrial genome, supporting the hypothesis that, similarly to nuclear epigenetics, mtDNA methylation could affect the expression of mitochondrial genes and mtDNA copy number as well [26,27,28,29,30]. Altered methylation at two regions of platelet mtDNA, the mitochondrially encoded Cytochrome C Oxidase I (*MTCO1)* and D-loop, have been previously associated with several pathological conditions, in particular cardiovascular [15] or metabolic diseases [31,32] respectively. Thus, considering the well-established role of platelets in CVD, investigating the association of TMAO with mtDNA methylation levels would help to clarify the picture describing the complex interplay between risk factors promoting cardiovascular events and the involved molecular pathways. Despite a certain consensus on the role of TMAO, there are still some concerns about considering l-carnitine intake as a risk factor directly associated with the promotion of cardiovascular events [33,34,35]. On the contrary, it has been demonstrated that, due to its ability to facilitate transport of long-chain fatty acids into the mitochondrial matrix, l-carnitine is able to trigger cardioprotective effects by reducing inflammation, oxidative stress and necrosis of cardiomyocytes [36]. Indeed, exogenous l-carnitine administration through dietary and intravenous routes has been suggested as an appropriate cardioprotective strategy [37,38].

This study aims to investigate, in a group of elderly women, (1) whether a 6-month l-leucine or l-leucine + l-carnitine supplementation combined with a resistance training protocol affects TMAO levels; (2) whether it affects methylation of mtDNA extracted from platelets in concordance with TMAO values; (3) the association of these parameters with a favorable/unfavorable blood lipid profile.

## 2. Results

### 2.1. Descriptive Statistics of Body Composition and TMAO Levels before Intervention

**Figure 1:**
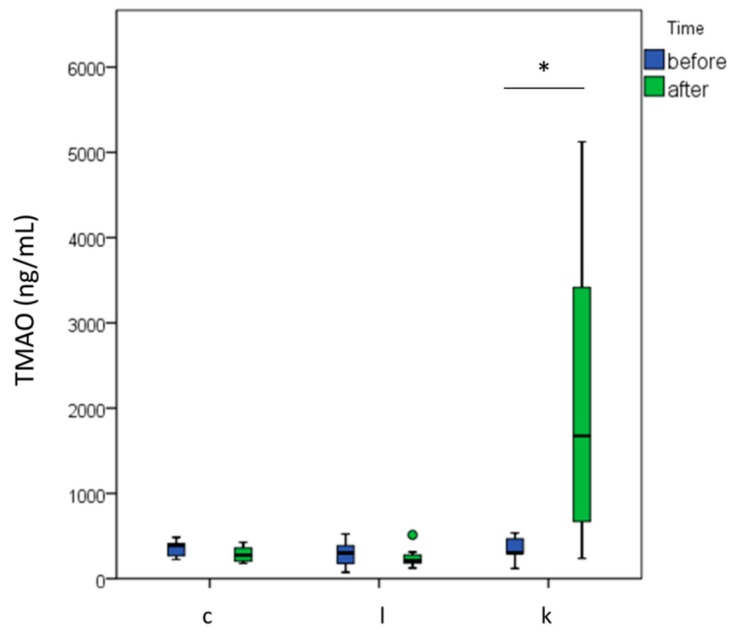
Trimethylamine-N-oxide (TMAO) levels in the intervention groups before and after the treatment. c = control group (*n* = 9); l = l-leucine group (*n* = 12); k = l-leucine + l-carnitine group (*n* = 9). * *p* < 0.05

Data were collected from a total of 30 women, from 62 to 72 years old. Descriptive statistics (Table 1) show that the mean BMI of the selected population was 28.0 ± 4.4. Data distribution for the variables describing body composition was normal (Table 1). Basal levels of TMAO were within a normal range [39] and they were normally distributed (Shapiro–Wilk, *p* = 0.628); no differences between the three intervention groups (controls, l-leucine group, l-leucine + l-carnitine group) were detected for TMAO measured before the intervention (ANOVA, *p* = 0.460) (Figure 1). 

### 2.2. Body Composition Parameters and Age Did Not Affect Basal TMAO Levels

Basal levels of TMAO (mean value 327.9 ± 123.9 ng/mL) did not correlate with any variable describing body composition (Basal Metabolic Rate, *p* = 0.944; Obesity Degree, *p* = 0.756; Visceral Fat Area, *p* = 0.697; Weight, *p* = 0.682; Skeletal Muscle Mass, *p* = 0.905; Body Fat Mass, *p* = 0.662; Percent Body Fat, *p* = 0.639; Body Mass Index, *p* = 0.757; *p* values referring to Pearson correlation test). Despite it has been established that TMAO increases with age [40], age did not affect basal TMAO levels in our group (Spearman correlation, *p* = 0.434). This result was expected considering that our population was highly homogeneous for sex and age (mean age 67.3 ± 2.7).

### 2.3. l-carnitine Supplementation Increased TMAO Levels after 6 Months of Supplementation and Physical Training

Data measuring TMAO after the intervention were not normally distributed (Shapiro–Wilk, *p* = 0.001). After the treatment, TMAO increased only in the group supplemented with l-leucine + l-carnitine respect to before the intervention (TMAO before = 344.3 ± 129.8 ng/mL; TMAO after = 2216.8 ± 1869.0 ng/mL; paired t-test, *p* = 0.02, paired samples Wilcoxon test, *p* = 0.021); no significant effects on TMAO were exerted by training alone (paired samples Wilcoxon test, *p* = 0.173) or l-leucine supplementation (paired samples Wilcoxon test, *p* = 0.388) after six months (Figure 1).

### 2.4. Lipid Profile after 6 Months of Supplementation and Training

No differences between groups at basal levels were observed for total cholesterol (TC) (*p* = 0.952), high density lipoprotein cholesterol (HDL-c) (*p* = 0. 868), low density lipoprotein cholesterol (LDL-c) (*p* = 0. 941), triglycerides (TG) (*p* = 0. 714), TC/HDL-c (*p* = 0. 824) and TG/HDL-c (*p* = 0. 641) (descriptive statistics available at Table S1). Lipid profile in the analyzed samples showed that neither the resistance training alone (paired t-test, *p* = 0.861) nor l-leucine (paired t-test, *p* = 0.479), nor l-leucine + l-carnitine (paired t-test, *p* = 0.338) affected total cholesterol levels. No significant changes were identified for LDL-c (in control group, *p* = 0.746; l-leucine group, *p* = 0.852; l-carnitine group, *p* = 0.283), TG (in control group, *p* = 0.861; L-leucine group, *p* = 0.479; l-carnitine group, *p* = 0.919), HDL-c (in control group, *p* = 0.336; l-leucine group, *p* = 0.973; l-carnitine group, *p* = 0.919); TC/HDL-c (in control group, *p* = 0.861; l-leucine group, *p* = 0.261; l-carnitine group, *p* = 0.317) or TG/HDL-c (in control group, *p* = 0.982; l-leucine group, *p* = 0.360; l-carnitine group, *p* = 0.886) ratios in all the intervention groups between the two time points of the study (Figure S1).

### 2.5. TMAO Levels after 6 Months Correlated with TC and LDL-c

**Figure 2:**
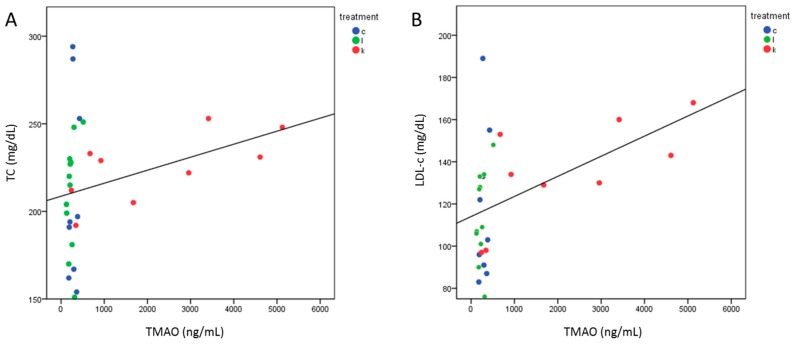
Correlation between TMAO levels and lipid profile after the intervention. The analysis shows a correlation between TMAO and total cholesterol (TC) (Spearman Rho = 0.407, *p* = 0.026) (**A**) or low density lipoprotein cholesterol (LDL-c) (Spearman Rho = 0.518, *p* = 0.003) (**B**). Treatments are indexed in different colors according to the legend: c = control group (*n* = 9); l = l-leucine group (*n* = 12); k = l-leucine + l-carnitine group (*n* = 9).

TMAO levels after 6 months were correlated with TC (Spearman Rho = 0.407, *p* = 0.026) (Figure 2A) and LDL-c (Spearman Rho = 0.518, *p* = 0.003) (Figure 2B) in all subjects. Repeating this analysis dividing the population by treatment, the correlation of TMAO was significant with LDL-c (Spearman Rho = 0.75, *p* = 0.02) and by trend with TC (Spearman Rho = 0.633, *p* = 0.06) only in l-leucine + l-carnitine group.

### 2.6. MtDNA Methylation Levels Analysis at MTCO1 and the D-Loop Region

As no changes for TMAO were observed in the control (i.e., 24 weeks resistance training without any supplementation) and l-leucine group, mtDNA methylation levels were primarily assessed in the control (*n* = 8) and l-leucine + l-carnitine group (*n* = 9), both before and after the intervention. In total, seven samples from l-leucine group (those with TMAO levels within the normal range) were analyzed as a secondary analysis only to exclude the effect of l-leucine on mtDNA methylation levels.

**Figure 3:**
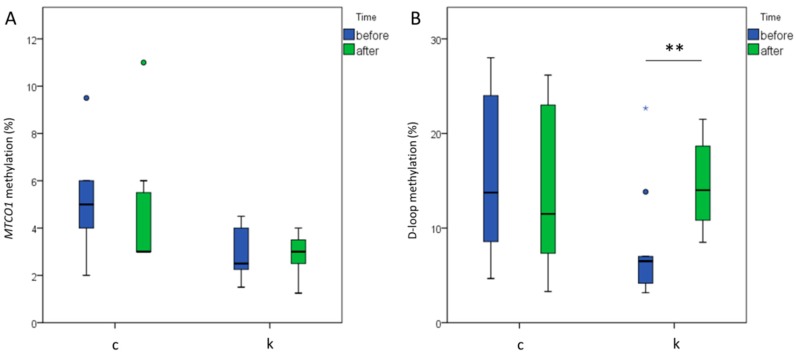
mtDNA methylation at *MTCO1* and D-loop in controls and treated with l-leucine + l-carnitine groups. No significant changes identified for time or treatment at *MTCO1* (**A**). Increased D-loop methylation levels (**B**) were detected at the D-loop region (paired t-test, *p* = 0.005). c = control group (*n* = 8); k = l-leucine + l-carnitine group (*n* = 9). Blue* defines outliers in the boxplot. black** define *p* < 0.01.

Analyses at *MTCO1* locus (whose methylation was previously associated with cardiovascular events [15]) showed low basal methylation levels (control group: mean % of methylation = 5.3 ± 2.4; l-leucine + l-carnitine group: mean % of methylation = 2.7 ± 1.9; no significantly different: Mann–Whitney-U test, *p* = 0.191), without changes after the intervention neither in the control (paired samples Wilcoxon test, *p* = 0.500) nor in the l-leucine + l-carnitine group (paired samples Wilcoxon test, *p* = 0.830) (Figure 3A).

On the other hand, remarkable methylation levels were detected at the analyzed area of the D-loop region. Basal mtDNA levels at D-loop (Shapiro–Wilk test, *p* = 0.007) did not correlate either with age (Spearman test, *p* = 0.897) or with lipid profile parameters (Spearman correlation: TC, *p* = 0.208; HDL-c, *p* = 0.368; LDL-c, *p* = 0.313; TG, *p* = 0.955; TC/HDL-c, *p* = 0.464, TG/HDL-c, *p* = 0.409). No significant differences for D-loop methylation were measured at basal levels between the control group (mean % of methylation = 15.6 ± 8.2) and l-leucine + l-carnitine (mean % of methylation = 8.1 ± 6.3) (Mann–Whitney-U test, *p* = 0.081) or between control and l-leucine group (mean % of methylation = 16.9 ± 10.4) (Mann–Whitney-U test, *p* = 0.458). D-loop methylation significantly increased after the supplementation with l-leucine + l-carnitine (+6.63%; paired t-test, *p* = 0.005), while no significant changes were induced by training alone (paired t-test, *p* = 0.616) (Figure 3B). As demonstrated by the secondary analysis, l-leucine alone was not able to induce the same increase after 6 months of supplementation in this sample (paired t-test, *p* = 0.201) (Figure S2); thus, l-carnitine is suggested to be responsible for the increased D-loop methylation measured in the studied group.

**Figure 4:**
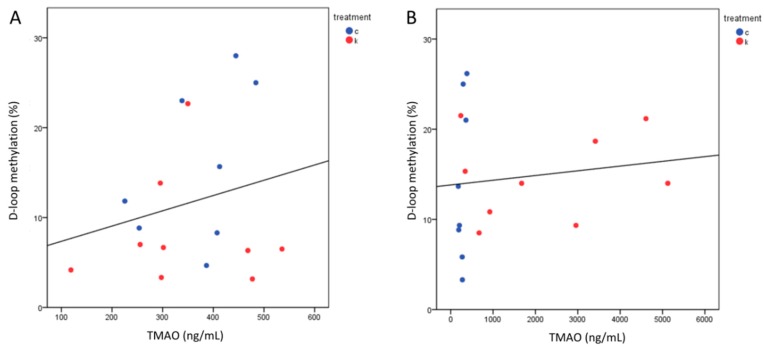
Correlation between TMAO and D-loop methylation before (**A**) and after (**B**) the intervention. Absence of a significant direct correlation between D-loop methylation and TMAO levels was observed in both the times. Treatments are indexed in different colors according to the legend: c = control group (*n* = 8); k = l-leucine + l-carnitine group (*n* = 9).

Since both higher D-loop methylation levels and increased TMAO values were measured in l-leucine + l-carnitine group, we tested the correlation between these two variables. Correlation between TMAO and D-loop methylation analyzing the totality of data was not significant (Spearman correlation, *p* = 0.436); no significant associations could be observed also analyzing data divided for treatments (Spearman correlation: controls, *p* = 0.135; l-leucine, *p* = 0.637; l-leucine + l-carnitine, *p* = 0.246) or time (Spearman correlation: before, *p* = 0.422; after, *p* = 0.337) (Figure 4).

### 2.7. D-Loop Methylation Inversely Correlated with TC and LDL in the Total Group of Subjects after 6 Months of Intervention

**Figure 5:**
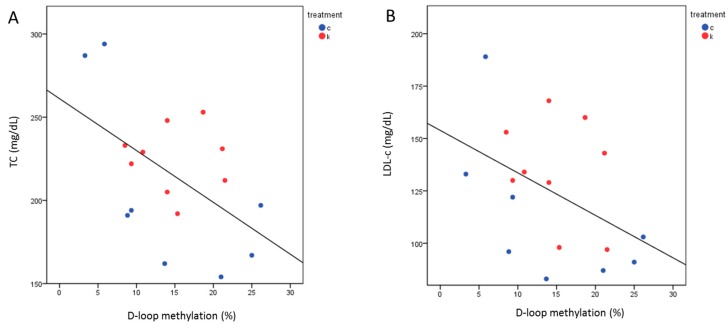
Correlation between D-loop methylation and LDL-c (**A**) or TC (**B**). Treatments are indexed in different colors according to the legend: c = control group (*n* = 8); k = l-leucine + l-carnitine group (*n* = 9).

Analyzing the correlation between DNA methylation at the D-loop region and lipid profiles measured after 6 months, we found that D-loop methylation inversely correlated with TC (Pearson coefficient = −0.529, *p* = 0.029) and LDL-c (Pearson coefficient = −0.439, *p* = 0.048) (Figure 5). No significant association with D-loop methylation could be detected after the intervention for the other parameters describing the lipid profile (Pearson correlation: HDL-c, *p* = 0.394; TG, *p* = 0.119; TC/HDL-c, *p* = 0.431, TG/HDL-c, *p* = 0.677). These data suggested that the increased level of D-loop methylation was associated with improvement of LDL-c and TC levels during the training intervention.

### 2.8. Increased D-Loop Methylation Was Associated with a Better Lipid Profile for HDL and TG in the l-leucine + l-carnitine Group

**Figure 6:**
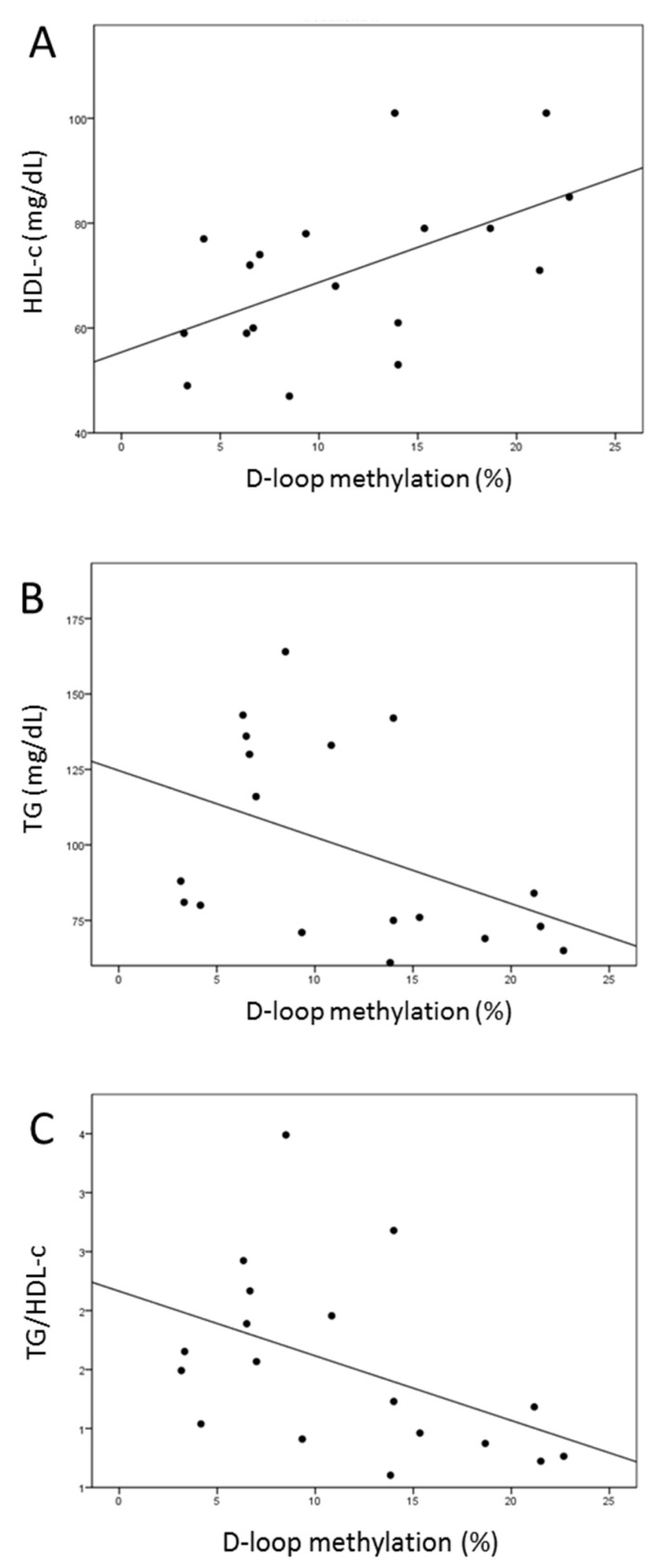
Correlations between D-loop methylation and lipid profile in the l-leucine + l-carnitine group. Correlation between D-loop methylation and high density lipoprotein cholesterol (HDL-c) (**A**), triglycerides (TG) (**B**), TG/HDL-c (**C**) before and after the intervention (*n* = 18) are presented.

In the l-leucine + l-carnitine group, increased D-loop methylation levels were correlated with higher HDL-c (Pearson coefficient = 0.55, *p* = 0.018) (Figure 6A) and lower TG (Spearman Rho = −0.488, *p* = 0.04) (Figure 6B). A by-trend correlation was observed also with reduced TG/HDL-c ratio (Pearson coefficient= −0.451; *p* = 0.06) (Figure 6C). No significant association between D-loop methylation and LDL-c was measured (Pearson coefficient= −0.92; *p* = 0.717; Figure S3).

### 2.9. Prediction Models for TMAO and D-Loop Methylation Increase

**Figure 7:**
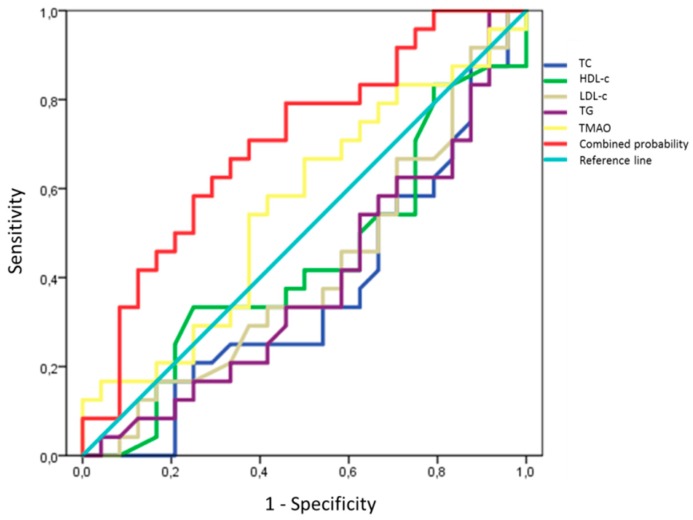
Receiver operating characteristic (ROC) curves predicting increased DNA methylation. TC, HDL-c, LDL-c, TG or TMAO alone are not able to significantly improve the prediction of increased D-loop methylation respect to the reference line. On the contrary, the combined predicted probability calculated according to a binary logistic regression model (including TC, HDL-c, LDL-c, TG and TMAO) significantly predicts the increase in D-loop methylation in our sample (auc = 0.703; *p* = 0.016).

A receiver operating characteristic (ROC) curve analyses testing the ability of lipid profile and TMAO to predict increased D-loop methylation (Figure 7) showed that none of the analyzed variables alone were able to significantly predict increasing in D-loop methylation (TC, auc = 0.365, *p* > 0.05; HDL-c, auc = 0.435, *p* > 0.08; LDL-c, auc = 0.415, *p* > 0.05; TG, auc = 0.391, *p* > 0.05; TMAO auc = 0.559, *p* > 0.05); on the contrary, only the binary logistic model including all the variables together was able to significantly increase the prediction given by the model (auc= 0.703; *p* = 0.016). This evidence led to the hypothesis that, since D-loop methylation was predicted by the combination of all the lipid profile variables and TMAO, this marker could be impacted by both the lipid profile and TMAO levels of the subjects.

## 3. Discussion

In recent years, numerous studies recognized the TMAO as an enhancer of cardiovascular risk via atherosclerotic lesion development [5,6,7]. TMAO has been associated not only with CVD but also with Alzheimer’s disease [41], metabolic syndrome [42], and cancer [43], which are all mediated by inflammatory processes. Although it has not been completely elucidated how it is related to CVD onset, there is a growing interest in TMAO [44] as a new molecular biomarker of CVD and as a potential therapeutic target [43,45]. In this study, we confirmed that l-carnitine supplementation increases TMAO levels, even if supplemented subjects performed regular physical activity. In accordance with previous studies identifying TMAO as a risk factor for cardiovascular events [5,10], in our subjects, the increase in TMAO was mildly associated with an unfavorable lipid profile (augmented TC and LDL). Nevertheless, it must be recognized that the usage of TC and LDL-c levels alone have intrinsic limits as CVD markers [46,47,48].

Since phosphatidylcholine/choline and/or l-carnitine are important sources of TMA via the gut microbiota [5,6,7], it has been postulated that the consumption of these TMA precursors (i.e., red meat) might be a critical factor promoting the risk of CVD development [49]. In contrast with this hypothesis, numerous studies investigating the biological effects of these nutrient supplementations did not support their positive association with CVD [50], suggesting that the relationship between nutritional intake of choline and l-carnitine and CVD could be more complex than as initially hypothesized. Moreover, a conspicuous body of literature suggests beneficial properties for l-carnitine intake [51] against metabolic diseases, including ischemic heart disease and skeletal muscle insulin resistance [52]. Fukami and collaborators, for example, demonstrated that oral l-carnitine supplementation might exert beneficial effects on vascular injury in hemodialysis patients, despite it was associated with higher TMAO levels [53]. Additionally, it has also been demonstrated that supplementation with l-carnitine could increase TMAO but it does not affect inflammatory [35] nor oxidative stress [54] markers in humans. This is coherent with the evidence supporting the usage of l-carnitine as a protective strategy against heart disease [36]. The hypothesis, suggesting a non-linear association among choline and l-carnitine intake, TMAO and CVD, is also supported by the observation that, despite fish is an important source of TMAO [55], its consumption is associated with positive effects on cardiovascular health [56,57].

Even though l-carnitine consumption increased TMAO levels in our group, new interesting evidence emerged analyzing platelets mtDNA methylation in the subjects supplemented with l-carnitine. First of all, significant levels of DNA methylation were identified in the analyzed samples, in particular in the regulatory D-loop region. D-loop is a particularly interesting region of the mtDNA because it regulates both mtDNA replication and transcription [58]. It is among the most methylated sites in the mtDNA, and its methylation has been associated with a reduction in mtDNA content, a potential marker of mitophagy, possibly suggesting that alterations in D-loop methylation may lead to changes in mitochondrial biogenesis [59]. Despite the fact that mtDNA methylation has been associated with mitochondrial gene transcription [60,61,62], there are some concerns about the possibility that D-loop methylation could regulate gene expression like in the nuclear DNA [27,63]. Other interesting evidence concerns the *MTCO1* gene, encoding for the subunit 1 of the cytochrome c oxidase, whose activity is associated with the electron transport chain complex. Genetic mutations in genes codifying for the cytochrome c oxidase are related to fatal metabolic disorders [64] which particularly affect tissues and organs with high energy demand, such as the heart. Additionally, a lower platelet cytochrome c oxidase activity is associated with sepsis mortality [65] (that frequently take place in the presence of cardiac dysfunction). Moreover, alterations of the expression of the cytochrome c oxidase complexes are well-known risk factors for severe mitochondrial diseases [64]. Interestingly, a remarkable increase in *MTCO1* methylation (+18%) has been measured in mtDNA from platelets of CVD patients [15], suggesting that alterations related to this gene could be implied in CVD pathogenesis. However, the evidence in support of this hypothesis are limited; thus, we decided to study this area to further investigate the potential implication of *MTCO1* in CVD pathogenesis.

While Baccarelli and collaborators [15] reported that mtDNA methylation levels in the *MTCO1* gene are higher in CVD patients than in healthy controls, we did not identify any significant change in *MTCO1* methylation after the supplementation. If we assume the increased methylation of *MTCO1* in platelets as a CVD biomarker [15], we can assert that l-leucine + l-carnitine supplementation did not worsen the CVD risk profile from the point of view of this biomarker. On the other hand, we demonstrated that l-carnitine supplementation was able to increase methylation in the D-loop region of platelets mtDNA significantly, and these increased levels were associated with an improved lipid profile (increased HDL-c, reduced TG) in the same group. No other studies investigated mtDNA methylation following training and l-carnitine supplementation, while previous evidence suggested positive effects of training [66,67] and l-carnitine [68] on platelets functions. Moreover, L-carnitine has a fundamental importance for cells and mitochondrial functionality [51], and thus it has a pivotal role for the cardiovascular system health [69]. Furthermore, it should be highlighted that not only mitochondrial genomics can play a relevant role (i.e., mutations of mitochondrial DNA can result in hypertension, atherosclerosis, and cardiomyopathy [70]), but also mtDNA copy number. Indeed, in 3 large prospective studies mtDNA copy number has been independently associated with CVD incidence, suggesting a potential clinical utility in improving CVD risk classification by this marker [71]. Despite therapies targeting mitochondria in the context of CVDs are not under widespread clinical use, there is a growing interest toward new strategies involving mitochondrial biology in early prevention or treatment of CVD [72].

Although, in our study, both TMAO and D-loop methylation increased after supplementation with l-carnitine, and despite TMAO has been previously shown to interact with platelets directly (increasing their responsiveness to thrombin and collagen, enhancing thrombosis potential in vivo) [14], we did not observe any direct correlation between platelets D-loop methylation and TMAO elevation after l-carnitine supplementation. On the contrary, the increase in D-loop methylation was associated with improved lipid profile in studied subjects. It is important to mention that a moderate TMAO increase has been previously demonstrated to be able to induce positive effects on the cardiovascular system [53,73]. Moreover, it has been hypothesized that the association of TMAO and CVD might be actually due to the abundance of TMA-producing bacteria in the gut, rather than to the direct effect of circulating TMAO (which could be indirectly associated and likely to be the result of dysbiotic gut microbiota) [74]. In addition, ROC curves analysis shows that increased D-loop methylation is predicted by the binary logistic model that includes variables describing lipid profile (TC, HDL-c, LDL-c, TG) and TMAO together. This suggests that D-loop methylation could be a predictor of both the lipid profile and the TMAO levels.

Nevertheless, additional studies with different designs (i.e., in CVD patients vs. healthy individuals) would help to elucidate causal and mechanistic explanations underlying the identified associations among the analyzed variables. Although this is a pilot study, it must be underlined that it is randomized trial which compared the same subjects at different time points; thus, the experimental design increases the reliability of the measured variations. However, despite statistical analysis clearly identified a variation of D-loop methylation comparing the same individual measured before and after the supplementation, a significant heterogeneity of D-loop methylation measurements can be observed among the controls. Thus, since the small sample size is a clear limitation of this study, further investigation in wider populations are needed to confirm this preliminary evidence and to validate this proposed biomarker in clinical practice. Moreover, our study involved only female Caucasian subjects. This is an advantage as the effect of l-carnitine supplementation has been investigated in a highly homogeneous population (for gender, age and ethnicity). However, these investigations should be replicated and confirmed in populations of different ethnicity and in males, particularly considering that mitochondria have been associated with marked sexual dimorphism (differential resistance to oxidative stress, oxidative capacities, and mitochondrial functions regulated by sex hormones [75]). Moreover, since the TMAO increase was limited to a period of 6 months, we cannot exclude that different effects could be observed in conditions of chronic TMAO elevation.

Although the acknowledged limitations of this study, the important role that nutritional supplements can have in CVD prevention is confirmed. As previously reviewed [76,77], nutritional supplements can play relevant roles in lipid levels control, thus reducing cardiovascular risk of dyslipidemic individuals. Several mechanisms through which nutraceuticals can improve dyslipidemia have been identified [76], but this paper highlights for the first time a new one potentially involved: mitochondrial DNA methylation. The research field investigating how functional foods and nutraceuticals can prevent CVD is growing and it is going to have a relevant clinical impact by understanding how to properly overcome lipid disorders consequences beyond common drug use.

## 4. Materials and Methods

### 4.1. Subjects

Subjects originally participated in the study aiming for the evaluation of l-carnitine in combination with l-leucine supplementation and resistance training on skeletal muscle function (ClinicalTrials.gov identifier: NCT03907592).

### 4.2. Human Experimental Protocol

Healthy volunteers from 60 to 72 years old were considered eligible for this intervention study. Individuals with CVD, liver and kidney diseases, gastrointestinal disorders (including stomach ulcers and erosions), neuromuscular disease, diabetes and other severe chronic diseases were excluded during the recruitment process. Additionally, subjects had to present a medical doctor’s corroboration about the lack of restrictions to perform resistance exercises before starting the exercise training program. The recruited subjects were randomly allocated and assigned to one of the different experimental groups. The study protocol has been approved by the Independent Bioethics Commission for Research at Medical University of Gdansk (NKBBN/354-201/2017). Before starting the experimental procedure, all subjects were informed about the treatment procedure, risks and expected outcomes and gave their written informed consent.

### 4.3. Resistance Exercise Training

Over a period of 24 weeks, all subjects participated in a resistance training program (twice a week) according to the protocol described previously [78]. Approximately 1 week prior to the experimental trial day, participants performed a familiarization session and one-repetition maximum (1RM) strength testing was also performed to determine the experimental exercise load. Resistance exercise training (RET) was performed twice a week on non-consecutive days for 24 weeks under direct supervision of a research assistant. The RET sessions began with a 5 min warm up on the treadmill (walk), and thereafter subjects performed 3 sets of 4 separate exercises in the following order: leg press, chest press or lateral pull-down, horizontal row or shoulder press, and leg extension. Chest press and horizontal row were only performed on one day, and lateral pull-down and shoulder press were only performed on the other day of the week (leg press and leg extension were performed at every RET session). The RET sessions were concluded with a 5 min cool down on a cycle ergometer and static stretching. During the first two weeks, training consisted of 3 sets of 10–12 repetitions and exercise load was kept at 65% of 1RM; after this period training consisted of 3 sets of 6–8 repetitions and was progressed to 80% of 1RM. The 1RM was re-evaluated every 2 weeks, and the training load was adjusted accordingly.

### 4.4. Supplementation

The control group participated in the training protocol without any supplementation, whereas two other groups were supplemented with either 4000 mg l-leucine per day or 1000 mg l-carnitine- l-tartrate in combination with 3000 mg l-leucine per day throughout the study period. The training procedure has been considered successful in participation at minimum 80% of exercise workouts and supplementation protocol have been completed by 37 subjects (control *n* = 12; l-leucine *n* = 13; l-leucine + l-carnitine *n* = 12).

### 4.5. Samples Collection

Fasting blood samples were taken from the antecubital vein before and after 24 weeks of the study interventions. Whole blood EDTA samples were centrifuged at 200× *g* at 4 °C for 15 min to obtain platelet-rich plasma (PRP). PRP was further centrifuged at 2000× *g* at 4 °C for 10 min. Platelets and plasma samples were separated and stored at −80 °C for later analyses. Due to technical problems platelets were obtained from control *n* = 9, l-leucine *n* = 12, l-leucine + l-carnitine *n* = 9. A flow-chart of analyzed samples is provided in supplementary materials (Figure S4).

### 4.6. Body Composition Assessment

Body mass and composition were estimated using a bioelectrical impedance analyzer (InBody720, InBody Co., Ltd., Seoul, Korea). The participants had emptied their bladders and bowels prior to the measurement. The analyses were performed in the position recommended by the manufacturer’s guidelines and the subjects clad only underwear. The “InBody720” measures the impedance of five segments of the body (each arm, each leg, trunk) at frequencies of 1, 5, 50, 250, 500, and 1000 kHz through the 8 polar tactile-electrode. Based on height, weight, age and the impedance, basal metabolic rate, obesity degree, visceral fat area, skeletal muscle mass, body fat mass and percent body fat were calculated [79].

### 4.7. TMAO Assessment

Plasma TMAO (ng/mL) was determined by the UPLC/MS/MS method in the Mass Spectrometry Laboratory, Institute of Biochemistry and Biophysics, Polish Academy of Sciences (Warsaw, Poland) as described previously [80].

### 4.8. Lipid Profile Assessment

TC, HDL-c and TG have been determined in serum samples using standard automatic analyzer Cobas6000 (Roche Diagnostics, Mannheim, Germany). According to Knopfholz and colleagues [81], the Friedewald formula (FF) was used to estimate LDL-c levels using TC, TG and HDL cholesterol.

### 4.9. DNA Extraction and Bisulphite Conversion

DNA was extracted from the frozen platelet pellets from 26 samples (9 controls, 8 l-leucine and 9 l-leucine + l-carnitine). Since the l-leucine group is an additional control and l-leucine alone is not able to increase TMAO levels, 4 subjects that displayed abnormal TMAO levels (out of 200–400 ng/mL [39]) were considered outliers and were not processed for methylation analysis. The DNA extraction was performed using the Genomic DNA extraction kit (Norgen Biotek Corp., Thorold, ON, Canada) according to manufacturer instructions. According to Baccarelli and collaborators [15], as platelets do not have nuclei, the extracted DNA is mitochondrial. Contamination for nuclear DNA was tested using specific primers that amplify nuclear and mitochondrial genes (Table S2) by q-PCR. No amplification products were obtained for nuclear genes (Ct detection > 30 or undetectable). Up to 500 ng of DNA was converted with bisulfite using the EZ DNA Methylation-Gold Kit (Zymo Research, Irvine, CA, USA) following the manufacturer protocol.

### 4.10. Measurement of mtDNA Methylation

Converted DNA was amplified for the two selected regions (*MTCO1*, D-loop) with specific primers [15,82] (Table S2) using the PyroMark PCR Kit (Qiagen, Hilden, Germany). Amplified products were visualized on an agarose gel at 1.2% to assess the specificity of the PCR reaction. Amplicons were sequenced using Pyromark Q24 instrument (Qiagen, Germany) according to the manufacturer’s protocol. Efficiency of bisulphite conversion was assessed according to the PyroMark Q24 protocol through internal controls. Data were further analyzed only if a complete conversion was assessed. The experiment was performed in technical duplicates. Values of methylation percentage were calculated for every single CpGs. As there were no significant different trends between the CpG in the same amplicon, a global mean % value for each analyzed sequence (*MTCO1*, D-loop) was calculated. Due to technical issues, 24 subjects (controls = 8; l-leucine = 7; l-leucine + l-carnitine = 9) were considered for the final analysis on mtDNA methylation.

### 4.11. Statistics

The Shapiro–Wilk test was used to analyze data distribution. A paired t-test was used to check for differences between groups at the two time points (before and after the treatment). The Pearson or Spearman correlation test was used to identify correlations between data when data were normally or not normally distributed, respectively. ANOVA or Kruskal–Wallis was used to test for differences among groups for basal levels of the analyzed clinical variables. Bonferroni’s correction was applied in case of multiple comparisons. ROC curves were calculated assuming increased values D-loop methylation over the median value (as this variable is not normally distributed, Shapiro–Wilk test, *p* = 0.017) of the analyzed group. The combined predicted probability was calculated according to a binary logistic regression model. Statistics and plots were realized using SPSS software. A post-hoc power analysis was performed using G*Power 3 [83] Software in groups where methylation changes due to the supplementation was detected (Figure S5). A *p* value ≤ 0.05 was considered as significant through the entire study.

## 5. Conclusions

The TMAO increase due to l-carnitine supplementation is mildly associated with a worse lipid profile even in an elderly female population undergoing regular resistance training. l-carnitine supplementation increases D-loop methylation in platelets, but this cannot be directly correlated to the TMAO increase observed in the same group. D-loop is confirmed as among the most responsive areas in terms of methylation variations due to environmental exposure; its increase predicts a more favorable lipid profile, corroborating the hypothesis that l-carnitine supplementation can exert positive effects on the cardiovascular system despite the induced TMAO elevation. Hence, our study supports the hypothesis that the correlation between TMAO and atherosclerosis might be more complex than already postulated and that alteration of mtDNA methylation in platelets (whose dysfunctions have been already associated with CVD [84,85]) could be involved in the pathogenesis of this multifactorial disease. Although a mechanistic explanation for the role of mtDNA methylation is still missing and a limited number of evidence on this topic exists, this pilot study supports the hypothesis that mtDNA methylation could be an interesting potential biomarker of both exposure and disease; indeed, it is important to highlight this new research field and its potential application in order to promote further investigations that could help to unveil the involved pathways and substantiate its usage in clinical practice. The growing research field of mitochondrial epigenetics shows, in particular, an interesting potential in CVD prevention, especially in association with other existing biomarkers whose meaning is still under investigation. Further studies in wider populations are necessary to validate the usage of l-carnitine supplementation in clinical practice.

## Figures and Tables

**Table 1:** Descriptive statistics for body composition before the intervention in the analyzed group. Normality of distribution was verified by the Shapiro–Wilk test and *p* values are shown for each variable.

	Mean ± SD	Range	Shapiro–Wilk (*p* Value)
Height (cm)	159.9 ± 5.2	151–175	0.241
Age (years)	67.3 ± 2.7	62–72	0.284
Basal Metabolic Rate	1342 ± 98	1183–1559	0.500
Obesity Degree	130.3 ± 20.7	90.8–165.0	0.075
Visceral Fat Area (cm^2^)	107.3 ± 36.4	40.5–170.8	0.268
Weight (kg)	71.9 ± 13.1	50.6–93.3	0.104
Skeletal Muscle Mass (kg)	24.6 ± 2.7	20.2–30.0	0.434
Body Fat Mass (kg)	26.9 ± 10.5	9.9–46.8	0.271
Body Fat (%)	36.1 ± 8.5	19.5–51.5	0.555
Body Mass Index	28.0 ± 4.4	19.5–35.5	0.076

## Supplementary Materials

The following are available online at https://www.mdpi.com/1422-0067/21/3/1047/s1.

## Abbreviations

1RM	One-repetition maximum
CVD	Cardiovascular disease
HDL-c	High-density lipoprotein cholesterol
LDL-c	Low-density lipoprotein cholesterol
*MTCO1*	Mitochondrial cytochrome c oxidase subunit 1 gene
mtDNA	Mitochondrial DNA
ROC	Receiver operating characteristic
TC	Total cholesterol
TG	Triglycerides
TMAO	Trimethylamine-N-Oxide
