# Supplementary material for: A Pilot Study on the Effects of l-Carnitine and Trimethylamine-N-Oxide on Platelet Mitochondrial DNA Methylation and CVD Biomarkers in Aged Women
Source: Int J Mol Sci. 2020 Feb 5;21(3):1047. doi: 10.3390/ijms21031047 (PMC7037757; doi:10.3390/ijms21031047)
Supplement: Supplementary file 1 [file ijms-21-01047-s001.pdf]

Supplementary Materials

Descriptive statistics							
Time	treatment		N	Min	Max	Mean	SD
Before	control	TMAO (ng/mL)	9	225.3	484.0	357.7	91.3
		TC (mg/dL)	9	157.0	278.0	208.4	48.3
		HDL-c (mg/dL)	9	45.0	144.0	71.7	29.7
		LDL-c (mg/dL)	9	77.0	187.0	113.4	41.2
		TG (mg/dL)	9	58.0	241.0	117.7	60.3
		TC/HDL-c	9	1.9	4.7	3.1	0.9
		TG/HDL-c	9	0.8	5.4	2.0	1.5
	L-leucine + L-carnitine	TMAO (ng/mL)	9	118.9	535.6	344.3	129.8
		TC (mg/dL)	9	132.0	296.0	209.0	44.4
		HDL-c (mg/dL)	9	49.0	101.0	70.7	15.9
		LDL-c (mg/dL)	9	67.0	199.0	118.7	38.6
		TG (mg/dL)	9	61.0	143.0	100.0	31.5
		TC/HDL-c	9	2.1	4.0	3.0	0.7
		TG/HDL-c	9	0.6	2.4	1.5	0.6
	L-leucine	TMAO (ng/mL)	12	74.2	523.8	293.2	141.1
		TC (mg/dL)	12	163.0	240.0	204.2	25.8
		HDL-c (mg/dL)	12	44.0	101.0	66.8	19.5
		LDL-c (mg/dL)	12	71.0	156.0	114.3	24.5
		TG (mg/dL)	12	45.0	246.0	115.4	53.1
		TC/HDL-c	12	1.9	4.2	3.3	0.8
		TG/HDL-c	12	0.5	4.9	1.9	1.2
After	control	TMAO (ng/mL)	9	178.7	426.6	287.2	88.2
		TC (mg/dL)	9	154.0	294.0	211.0	53.5
		HDL-c (mg/dL)	9	42.0	131.0	69.1	26.5
		LDL-c (mg/dL)	9	83.0	189.0	117.7	35.8
		TG (mg/dL)	9	78.0	177.0	121.4	29.4
		TC/HDL-c	9	2.2	4.0	3.2	0.6
		TG/HDL-c	9	0.9	3.1	2.0	0.8
	L-leucine + L-carnitine	TMAO (ng/mL)	9	238.1	5124.5	2216.8	1869.0
		TC (mg/dL)	9	192.0	253.0	225.0	19.7
		HDL-c (mg/dL)	9	47.0	101.0	70.8	16.1
		LDL-c (mg/dL)	9	97.0	168.0	134.7	24.9
		TG (mg/dL)	9	69.0	164.0	98.6	36.9
		TC/HDL-c	9	2.1	5.0	3.4	0.9
		TG/HDL-c	9	0.7	3.5	1.6	1.0
	L-leucine	TMAO (ng/mL)	12	124.3	512.7	237.4	103.9
		TC (mg/dL)	12	151.0	251.0	210.3	30.7

		HDL-c (mg/dL)	12	47.0	107.0	72.6	18.9
		LDL-c (mg/dL)	12	76.0	148.0	116.0	21.2
		TG (mg/dL)	12	54.0	200.0	109.6	43.6
		TC/HDL-c	12	2.1	4.6	3.0	0.7
		TG/HDL-c	12	0.6	4.3	1.7	1.1

Supplementary Table 1. Descriptive statistics of TMAO and blood lipids in the population divided per groups and time of intervention.

**MTCO1**	TATTAATTGGTTTTTTAGGGTTTAT	Bio-CAACAAATCATTTCATAT TACTTC C	TATTTATAGTAGGAAT
**D-Loop**	GTGTATTGTTTTGAGGAGGTAAGT	Bio-CACTCCCATACTACTAATCTCATCAAT	TTTTTGGGGTTTGGT
**mtpair.21**	AATCCAAGCCTACGTTTTCACA	AGTATGAGGAGCGTTATGGAGT	
**mtpair.19**	AATCGAGTAGTACTCCCGATTG	TTCTAGGACGATGGGCATGAAA	
**GAPDH**	CTCTGCTCCTCCTGTTCGAC	AATCCGTTGACTCCGACCTT	
**AR**	TCCAGAATCTGTTCCAGAGCGTGC	GCTGTGAAGGTTGCTGTTCCTCAT	

Supplementary Table 2. Sequences of primers used for pyrosequencing of mtDNA and contamination test.

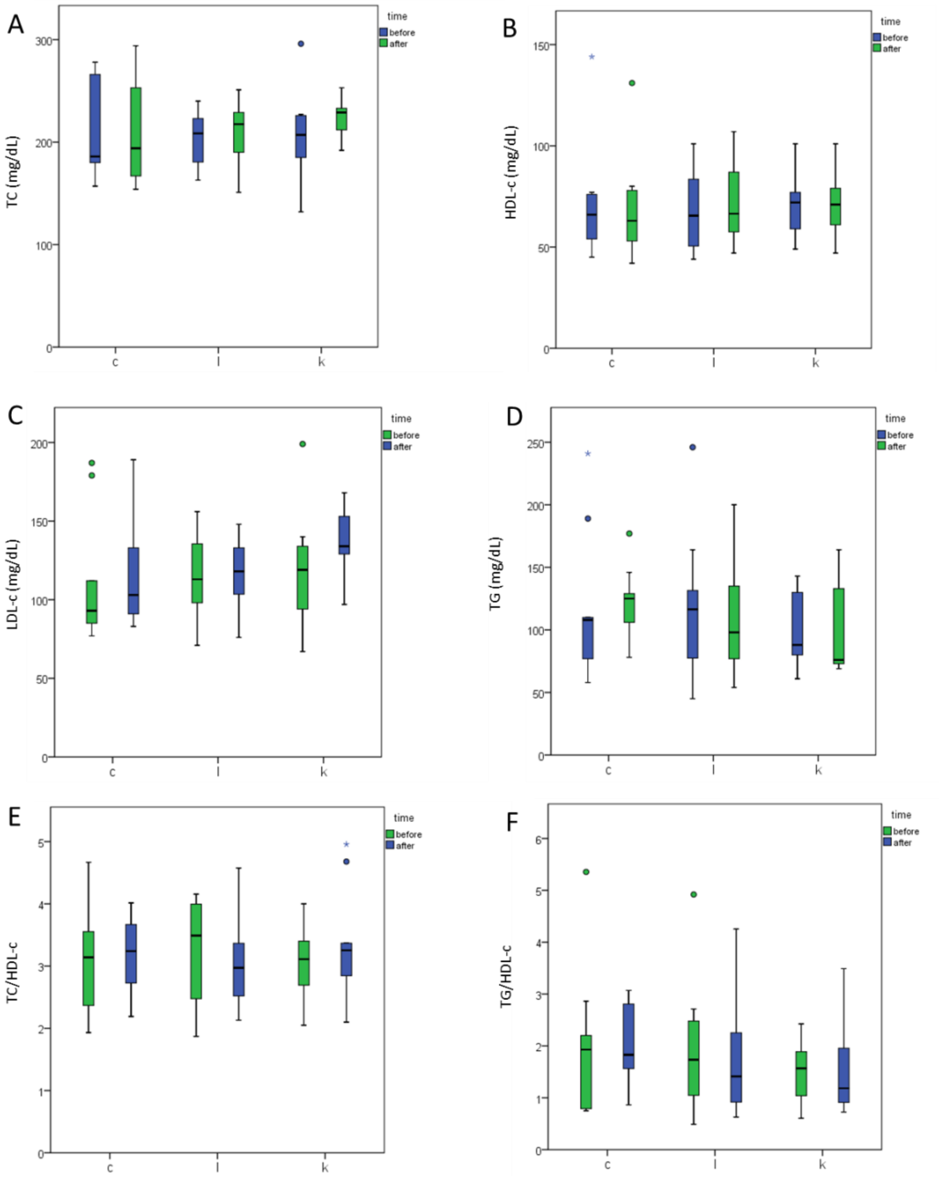

Supplementary figure 1. Lipid profile in the different intervention groups before and after the interventions. TC (A), HDL-c (B), LDL-c (C), TG (D), TC/HDL-c ratio (E) and TG/HDL-c ratio (F) before and after each intervention are displayed. c = control group (n=9); l = L-leucine group (n=12); k = L-leucine + L-carnitine group(n=9).

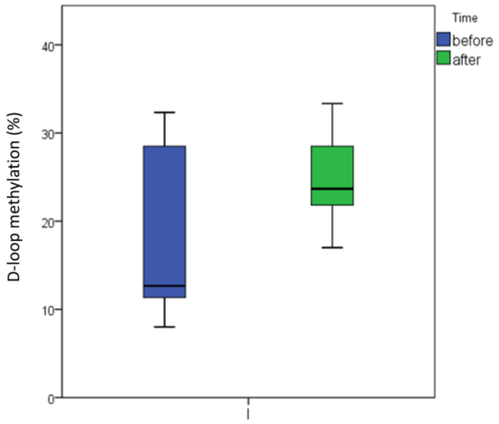

Supplementary figure 2. D-loop methylation in leucine group (n=7), before and after the treatment.

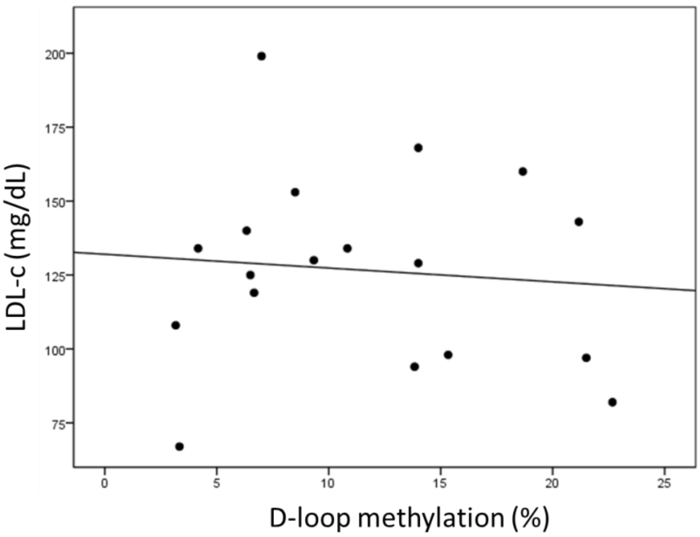

Supplementary figure 3. Correlations between D-loop methylation and LDL in the L-leucine + L-carnitine group (n=18). No significant association was measured (Pearson coefficient= -0.92; p= 0.717)

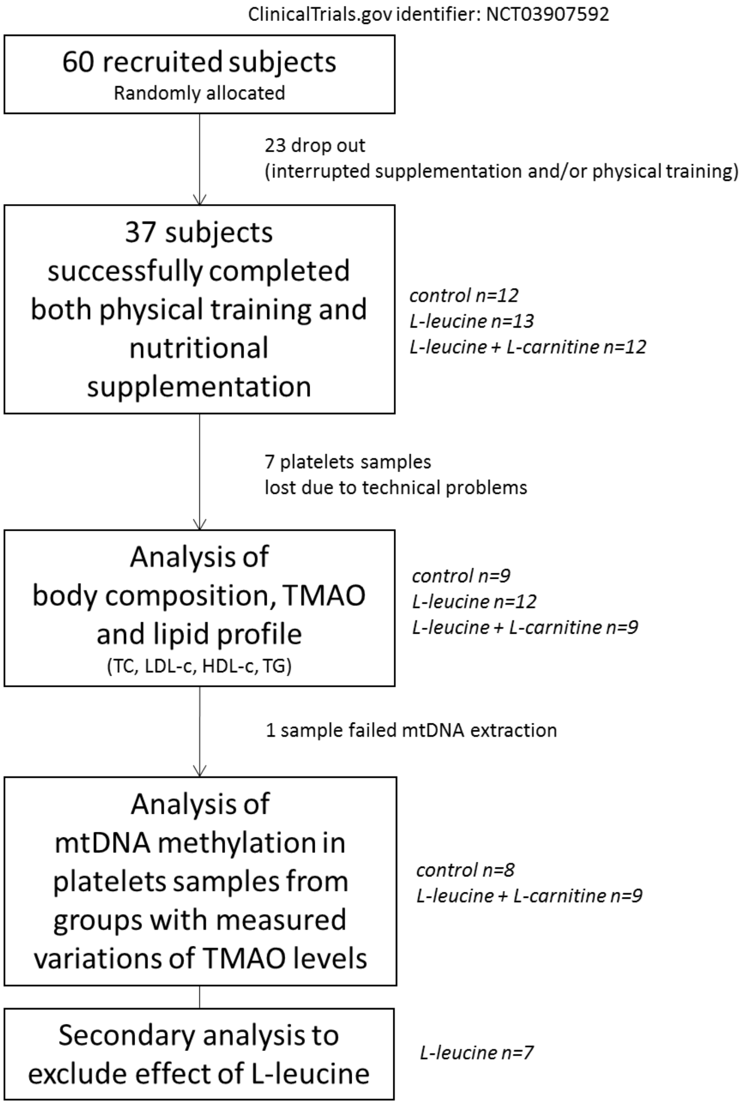

Supplementary figure 4. Flow-chart of the study. Graphical representation of the analyzed samples through the study.

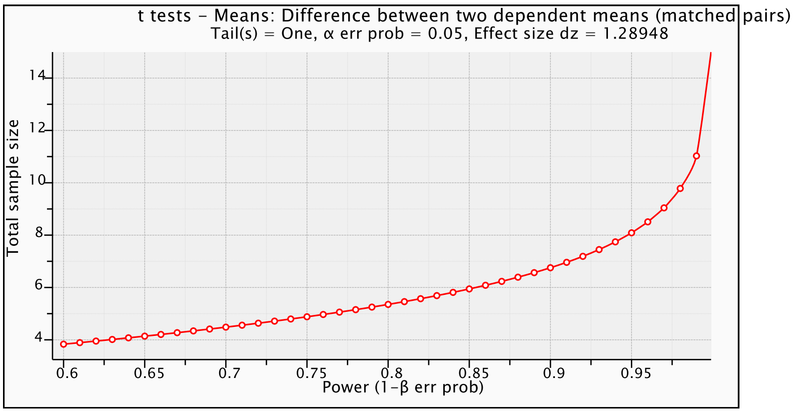

Supplementary figure 5. Power calculation analysis of D-loop methylation change in the L-leucine + L-carnitine group. The calculated effect size was 1.289; the actual calculated power was 0.969. When comparing the same group before and after the treatment, a sample size of 7 can guarantee a power of 0.9.
